# Combining best evidence: A novel method to calculate the alcohol-attributable fraction and its variance for injury mortality

**DOI:** 10.1186/1471-2458-11-265

**Published:** 2011-04-27

**Authors:** Benjamin J Taylor, Kevin D Shield, Jürgen T Rehm

**Affiliations:** 1 Social and Epidemiological Research Department, Centre for Addiction and Mental Health, Toronto, Ontario, Canada; 2 Dalla Lana School of Public Health, University of Toronto, Canada; 3 Department of Psychiatry, University of Toronto, Canada; 4 Epidemiological Research Unit, Technische Universität Dresden, Klinische Psychologie & Psychotherapie, Dresden, Germany

## Abstract

**Background:**

The alcohol-attributable fraction for injury mortality is defined as the proportion of fatal injury that would disappear if consumption went to zero. Estimating this fraction has previously been based on a simplistic view of drinking and associated risk. This paper develops a new way to calculate the alcohol-attributable fraction for injury based on different dimensions of drinking, mortality data, experimental data, survey research, new risk scenarios, and by incorporating different distributions of consumption within populations. For this analysis, the Canadian population in 2005 was used as the reference population.

**Methods:**

Binge drinking and average daily consumption were modeled separately with respect to the calculation of the AAF. The acute consumption risk was calculated with a probability-based method that accounted for both the number of binge drinking occasions and the amount of alcohol consumed per occasion. The average daily consumption was computed based on the prevalence of daily drinking at various levels. These were both combined to get an overall estimate. 3 sensitivity analyses were performed using different alcohol consumption parameters to test the robustness of the model. Calculation of the variance to generate confidence limits around the point estimates was accomplished via Monte Carlo resampling methods on randomly generated AAFs that were based on the distribution and prevalence of drinking in the Canadian population.

**Results:**

Overall, the AAFs decrease with age and are significantly lower for women than men across all ages. As binge drinking increases, the injury mortality AAF also increases. Motor vehicle collisions show the largest relative increases in AAF as alcohol consumption is increased, with over a 100% increase in AAF from the lowest to highest consumption category. Among non-motor vehicle collisions, the largest change in total AAF occurred both for homicide and other intentional injuries at about a 15% increase in the AAF from the lowest to the highest binge consumption scenarios.

**Conclusions:**

This method combines the best available evidence to generate new alcohol-attributable fractions for alcohol-attributable injury mortality. Future research is needed to refine the risk function for non-motor vehicle injury types and to investigate potential interactions between binge drinking and average volume of alcohol consumption.

## Background

The proportion of a disease or outcome that is due to the influence of some external causal factor is called the attributable fraction [1]. In alcohol epidemiology, this fraction is termed the alcohol-attributable fraction (AAF) and is defined as that proportion of disease that would disappear if alcohol consumption went to zero. In the categorical case [2], it has been calculated using the formula [1,3]:(1)

where P_i _represents the proportion of the population exposed in group i and RR_i _is the relative risk of mortality in exposed group i compared with the reference group (in alcohol often non-drinkers or lifetime abstainers). This is computed for as many drinking categories exist, from i = 0 to k, where i = 0 represents the reference group. This framework has been used extensively by the World Health Organization to estimate the burden of disease as a part of its Comparative Quantification of Risk analysis [4-6], and has been used by colleagues in other countries to establish the alcohol-attributable burden of disease [7-9].

However, these calculations have historically been relatively simplistic, with calculations usually being performed for three categories of average consumption only. More recently, a more differentiated consideration of average alcohol consumption has been introduced [10].

The calculation of AAF for injuries is a conceptually different than for chronic disease, since the acute effects of alcohol become very important and reliance on average consumption alone would considerably bias the results towards lower fraction estimates [11,12].

Recent work by this group has attempted to improve on the calculation of the AAF for injury by trying to account multiple drinking scenarios and by including other alcohol-drinking variables to better assess fatal injury risk [13,14].

This has meant incorporating 2 different dimensions of alcohol consumption for computing injury AAF: (1) drinking pattern measures such as binge drinking (both number of weekly occasions and the amount consumed per occasion) and (2) by additionally accounting for mean daily consumption of alcohol by modeling the specific distribution of drinkers and their daily drinking habits within a given population. What's more, we have tried to include alcohol metabolism rates in the liver to better assess time at risk of injury during intoxication, and, even more recently, attempting to account for the discrepancy between per capita consumption versus actual consumption in average daily alcohol drinking levels [15,16].

The end result of these attempts has been the incorporation of data from many different sources, making this AAF calculation a veritable "data melting pot" - it combines survey data, meta-analyses of relative risk, mortality data, and experimental lab data. While this is not problematic for the calculation of the AAF point estimate, it is very complicated for the calculation of the variance around each point estimate, as each source of data has its own distribution and variance, making combining their different errors complex.

This paper attempts a novel method (the distributional approach) developed by our group to more accurately calculate the AAF and its variance for injury mortality. The main objectives of this paper are four-fold:

1. Present the method to calculate alcohol-attributable fractions for fatal injury, its inherent sources and assumptions, and its data sources.

2. Present the point estimate and uncertainty estimates

3. Provide sensitivity analyses to provide context and alternative scenarios for the above

4. Discuss future improvements that will help in more accurate calculation of the AAF for mortality

## Methods

The approach we used to develop AAFs for injury mortality will be presented below following a brief description of the underlying survey, as it was the source of the alcohol consumption data, one of the most important driving factors behind both the AAF point estimate and the corresponding confidence interval.

### Description of underlying survey

For all alcohol consumption data used in the calculations (except for one of the sensitivity analyses), the Canadian Alcohol and Drug Use Monitoring Survey (CADUMS) 2008 [17] was used. It is a nationally representative survey of alcohol consumption in Canada and is representative of alcohol drinking in 2005. The precise methods used in the CADUMS are available elsewhere [18]. In brief, though, it was an 8-month long telephone survey that used random-digit dialing to identify respondents. The survey reported a response rate of 36.5%, with 15, 801 individuals in the final dataset. It was these individuals that provided binge drinking estimates and average daily consumption data for the distributional method.

#### A. Computing the probability of alcohol-attributable injury for a given drinking scenario

The method described here builds on earlier work by Taylor et al. [13] and Rehm et al. [14]. Briefly, it calculated the probability of dying from an alcohol-attributable injury from binge drinking and daily consumption separately, and then added each together to get a final probability of death for each injury as a function of total alcohol consumption (binge + average daily drinking). This resulting probability was then converted to numbers of deaths due to both binge and daily drinking, and finally divided by the total number of deaths from all causes to estimate the AAF for each injury subtype. All calculations calculated consumption variables using the Canadian standard drink definition (13.6 grams of pure alcohol). The method describes here uses the following inputs:

1. Mortality data for Canada for the year 2005 by age and sex for each injury subtype. Please see Table 1 for a list of the injuries considered in this analysis.

**Table 1:** Injury categories and the source of the relative risk relationship with alcohol consumption.

Condition	ICD 10 Code	Source for AAF Calculation
**Unintentional injuries**		
Motor vehicle collision	§	[13,14,20]
Poisonings	X40-X49	[13,14,20]
Falls	W00-W19	[13,14,20]
Fires	X00-X09	[13,14,20]
Poisonings and exposure to alcohol	X45	[13]
Drowning	W65-W74	[13,14,20]
Other Unintentional injuries	†Rest of V-series and W20-W64, W 75-W99, X10-X39, X50-X59, Y40-Y86, Y88, and Y89	[13,14,20]
**Intentional injuries**		
Self-inflicted injuries	X60-X84 and Y87.0	[13,14,20]
Intentional self-poisoning by and exposure to alcohol	X65	[13]
Homicide	X85-Y09, Y87.1	[13,14,20]
Other intentional injuries	†	[13,14,20]

§ V021-V029, V031-V039, V041-V049, V092, V093, V123-V129, V133-V139, V143-V149, V194-V196, V203-V209, V213-V219, V223-V229, V233-V239, V243-V249, V253-V259, V263-V269, V273-V279, V283-V289, V294-V299, V304-V309, V314-V319, V324-V329, V334-V339, V344-V349, V354-V359, V364-V369, V374-V379, V384-V389, V394-V399, V404-V409, V414-V419, V424-V429, V434-V439, V444-V449, V454-V459, V464-V469, V474-V479, V484-V489, V494-V499, V504-V509, V514-V519, V524-V529, V534-V539, V544-V549, V554-V559, V564-V569, V574-V579, V584-V589, V594-V599, V604-V609, V614-V619, V624-V629, V634-V639, V644-V649, V654-V659, V664-V669, V674-V679, V684-V689, V694-V699, V704-V709, V714-V719, V724-V729, V734-V739, V744-V749, V754-V759, V764-V769, V774-V779, V784-V789, V794-V799, V803-V805, V811, V821, V830-V833, V840-V843, V850-V853, V860-V863, V870-V878, V892.

†Rest of V = V-series MINUS §.

**Figure 1:**
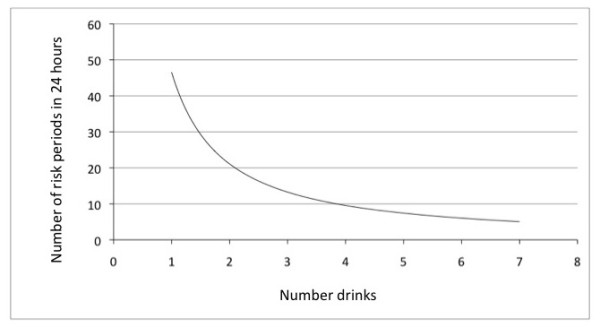
**Number of risk periods in a 24-hour period based on alcohol liver clearance rates, based on **[19].

2. The mean frequency of binge drinking (5+ drinks per occasion for men, 4+ drinks per occasion for women) occasions in the past year, by age and sex. This was calculated using the CADUMS database previously described. For average daily consumption, frequency was set to 365 (= every day).

3. The amount of alcohol consumed per occasion in grams of pure alcohol. For average daily consumption, this was the consumption by age and sex. Please see section B of these methods for the calculation of average daily consumption using the CADUMS 2008 data. For binge drinking, this quantity was estimated from the CADUMS data, which used 4+ and 5+ drinks per occasion. For the main analysis, 4 and 5 drinks for men and women, respectively, was used. Three different quantities were used for the sensitivity analysis.

4. Alcohol metabolism rates: the rate at which alcohol is metabolized by the liver must be accounted for in the adjustment of risk, since injury risk is only apparent as long as alcohol is exerting its effects. Therefore, the rate of alcohol clearance by the liver was modelled based on [19]http://pubs.niaaa.nih.gov/publications/aa35.htm and then converted into a risk period for a given number of drinks in a 24-hour time period. It corrects for the fact that, for one drinking occasion, the individual consuming a drink is not at risk for an entire 24-hour period on the day in which consumption occurs and varies by numbers of drinks consumed in one drinking occasion. As a result, higher numbers of drinks result in fewer (but longer) individual risk periods. For example, for three drinks consumed in one occasion, which carries a risk period of approximately 3 hours, there would be 8 (24/3) possible individual risk periods in a 24 hour period. On the other hand, for consumption of 1 drink, which carries a risk period of 30 minutes, there are consequently 48 separate possible risk period in 24 hours. Please see Figure 1 for the results of this modeling.

5. RR function: The relationship between the amount of alcohol consumed for one drinking occasion was determined via meta-analysis [20]. Fractional polynomial meta-regression was used to determine the best-fit line for each of motor-vehicle injury and non-motor-vehicle injury mortality (includes falls, fires, violence, drowning, poisoning, suicides, other intentional injury, and non-intentional injury combined), respectively. The final results of this meta-analysis were the following risk functions:(2a)(2b)

Where x = dose of pure ethanol (in grams) in one drinking occasion [20]

The curves become relatively unstable at levels beyond approximately 100 grams of pure alcohol per occasion due to a scarcity of data points beyond this level in any of the studies include in the original meta-analysis. Accordingly, if alcohol consumption per occasion was indicated as greater than 108 grams per day, the RR function was calculated based on exactly 108 grams per occasion.

#### B. Calculating average daily consumption

It is well known that population surveys underestimate adult per capita consumption [21]. This discrepancy between the estimated alcohol consumption of Canadians from survey and adult per capita consumption data arise from the fact that those excluded from the CADUMS 2008 consume more alcohol than the general population, such as the homeless, respondents not answering truthfully or having problems recalling the amount of alcohol consumed in the week prior to when they participated in the survey, and people who don't participate in surveys consuming more alcohol on average than people who do participate in surveys [16]. For the CADUMS 2008 we estimated undercoverage to be 27% (calculated by dividing the alcohol consumption estimated from per capita data by the alcohol consumed estimated from the CADUMS 2008). Thus, data on consumption from population surveys need to be triangulated with estimates of adult per capita consumption. Adult per capita consumption is based on sales import and export data and is generally considered to be the most accurate measure of consumption. To calculate the up-shifted mean daily consumption, we multiplied the sex and age specific means by the estimated under coverage of the CADUMS 2008; to be conservative, under coverage was calculated assuming that 10% of adult per capita consumption was not consumed.

The up-shifted daily alcohol consumption distribution was then calculated based on methods outlined by Kehoe and colleagues [22], who found that average daily alcohol consumption could be modeled using a gamma distribution. Furthermore, using regression analysis they found that the standard deviation of this distribution could be expressed empirically as a function of the mean. Based on this function, we were able to calculate the shape (θ) and scale (κ) parameters of the gamma distribution.

#### C. Calculation of the AAF

The formula to compute the alcohol-attributable injury for binge consumption is presented below:(3)

where P_abs+former _is the proportion of lifetime abstainers and former drinkers, and P_current(Binge) _and P_Current(Non-Binge) _are the prevalences of current drinkers who engage and who do not engage in binge drinking, respectively. RR_binge_(x) represents the risk ratio for binge drinkers given a binge amount of alcohol consumed corrected for both time at risk and number of drinking occasions. RR_binge_(x) was calculated as follows:(4)

where P_dayatrisk _(calculated based on the average binge consumption x) and P_daysatrisk _represent the proportion of a given day during which a person binge drinks and is at risk, and the percentage of days the person undertakes binge drinking, respectively.

The formula to compute the alcohol-attributable injury for average consumption is below:(5)

where P (x) represents the prevalence of drinking at level x (in grams per day, modeled by the gamma function. The RR(x) is the relative risk at this level compared to lifetime abstainers and former drinkers, corrected for time at risk. As average consumption is a daily intake estimate, no correction for the number of drinking occasions was needed. To adjust the RR(x) for time at which a person is at risk for an injury, we computed the time at risk through the modeling alcohol metabolism rates, namely, the rate at which alcohol is metabolized by the liver using the following formula:(6)

where P_dayatrisk _(calculated here based on the drinking level x) represents the proportion of a day at risk per drinking occasion, and RR_Crude_(x) is the relative risk at drinking level x compared to being sober, not adjusted for the time at risk per occasion.

#### C. Methods to calculate the uncertainty estimates

A Monte Carlo-like approach was used to calculate the 95% confidence intervals (CIs) of the AAFs for average and binge consumption [23]. First, we estimated the variance of the AAF from 10,000 randomly generated AAFs that were calculated from 10,000 random sets of the lowest level parameters (the parameters from which all other values are derived) for each age, sex and injury type. Parameters were generated based on their distribution, mean and variance. These parameters were then used to calculate the risk ratio functions, prevalence of drinkers, number of drinking occasions per year, time at which a person is at risk, and the amount consumed per occasion (expressed as a consumption prevalence distribution for average consumption and a point estimate for binge consumption).

##### Generating average consumption for AAF parameters

For the average consumption AAF, we generated estimates of under coverage by first generating the prevalence of current drinkers for each age and sex group. The average daily alcohol consumption among current drinkers for the population mean was then calculated based on the weighted average derived from group and sex specific prevalences and means. The coverage rate was then calculated by dividing 90% of the estimated per capita consumption by the generated population mean. Age and sex specific means were then up-shifted by multiplying these means by the inverse of the generated coverage rate.

The κ parameter of the gamma distribution was generated in accordance with Rehm and colleagues [10], while the θ parameter was calculated by dividing the generated up-shifted mean daily alcohol consumption by the generated κ parameter.

##### Generating binge consumption AAF parameters

Prevalence of binge drinkers was generated based on estimates derived from the CADUMS 2008. The numbers of binge drinking occasions were generated based on estimates derived from the National Epidemiologic Survey on Alcohol and Related Conditions data (2001 - 2002).

Risk ratio estimates for both binge and average consumption AAFs were generated based on the variance of the beta estimate from the fractional polynomial meta-regression.

#### D. Sensitivity Analysis

Sensitivity analyses were planned *a priori *to test the robustness of the methods to theoretical increases in binge drinking since this is a major driver, if not *the *major driver of alcohol-attributable injury and of this AAF estimation method. There are 3 sensitivity analyses planned, each showing increases in binge drinking quantity. This meant from the original 4/5 drinks (54.4 and 68 grams per drinking occasion) per occasion for men and women, respectively, this consumption level was increased to 5/6 drinks per occasion (Sensitivity Analysis I), and 6/7 drinks per occasion (Sensitivity Analysis II). Lastly, the average number of drinks per drinking occasion, by age and sex, was computed from the National Epidemiological Survey on Alcohol and Related Conditions (NESARC) 2001 and 2002 was used to simulate a "real life" scenario for Canadians, assuming, of course, that Canadians consumed approximately equally amounts to white Americans (Sensitivity Analysis III). Details of the NESARC and its methods, sampling frame, and questions can be found elsewhere [24,25].

All calculations and simulations were performed using R (version: 2.11.1).

## Results

Table 2 shows the average daily consumption estimates, by age and sex. This table shows that the majority of men and women in all age groups drink, but there are differences by age and sex. In general the age group 15-29 appears to have the highest consumption pattern, with decreases in 30-44 year olds and then another increase from 45-59 year olds. However, in the number of drinking occasions per week, the current Table 2 shows a steady increase from 15-29 years olds through older age groups and then finally decreasing in those aged 70 and older. More men drink compared to women, and drink more on average. What's interesting on this table also is the dramatic increases in mean alcohol consumption following the up-shift to correct for per capita consumption, at times approximately a 3-fold increase.

**Table 2:** Average daily alcohol consumption estimates for Canada, 2008.

		Raw estimates (current drinkers)		Corrected estimates (current drinkers)		Number of drinking occasions per week	
				
Gender	Age group	Mean (g/day)	95% CI	Mean (g/day)	95% CI	Mean number	95% CI
**Women**	15 - 29	9.0	(3.7-14.4)	27.9	(23.6-32.2)	0.81	(0.64-0.98)
	30 - 44	3.6	(3.1-4.1)	11.2	(10.5-11.9)	0.77	(0.67-0.87)
	45 - 59	4.9	(4.2-5.6)	15.2	(14.1-16.3)	1.07	(0.95-1.18)
	60 - 69	4.6	(3.8-5.5)	14.3	(13.0-15.6)	1.04	(0.84-1.23)
	70 - 79	4.4	(3.1-5.6)	13.5	(11.8-15.1)	0.91	(0.66-1.17)
	80+	4.1	(2.5-5.6)	12.6	(10.8-14.3)	0.86	(0.51-1.21)
**Men**	15 - 29	12.8	(10.1-15.6)	39.7	(36.0-43.4)	1.13	(0.94-1.31)
	30 - 44	9.7	(8.1-11.4)	30.1	(27.8-32.5)	1.35	(1.19-1.52)
	45 - 59	11.7	(9.3-14.2)	36.3	(33.9-38.8)	1.66	(1.47-1.84)
	60 - 69	11.1	(8.2-13.9)	34.3	(29.9-38.7)	1.65	(1.35-1.95)
	70 - 79	9.9	(7.0-12.7)	30.6	(25.9-35.2)	1.69	(1.24-2.14)
	80+	5.9	(3.2-8.6)	18.2	(15.0-21.3)	1.41	(0.83-1.99)

Table 3 shows how the RR value changes with the quantity consumed per binge occasion for the main analysis and each of the three sensitivity analyses. As alcohol consumption increases, the RR also increases. However, of particular note in this table is that for lower alcohol consumption values (4/5 drinks per occasion) the RR for MVA injury is lower than for non-MVA injury, but as alcohol consumption increases, the risk of an MVA injury surpasses that of a non-MVA injury due to the steeper dose-response curve of MVA injury, also highlighted by the steep increase in risk with modest increases in per-occasion consumption.

**Table 3:** Description of the relative risk of injury mortality by age and sex for the main analysis (4/5 drinks per occasion) and for each of the sensitivity analyses for motor vehicle injury and non-motor vehicle injury mortality.

	15-29		30-44		45-59		60-69		70-79		80+	
	M	W	M	W	M	W	M	W	M	W	M	W
**Motor Vehicle Injury**												
Main Analysis	4.58	2.65	4.58	2.65	4.58	2.65	4.58	2.65	4.58	2.65	4.58	2.65
Sensitivity Analysis I	8.96	4.58	8.96	4.58	8.96	4.58	8.96	4.58	8.96	4.58	8.96	4.58
Sensitivity Analysis II	19.77	8.96	19.77	8.96	19.77	8.96	19.77	8.96	19.77	8.96	19.77	8.96
Sensitivity Analysis III	43.57	8.75	19.30	7.84	19.30	7.84	10.61	6.85	10.61	6.85	10.61	6.85
**Non-Motor Vehicle Injury**												
Main Analysis	6.08	5.03	6.08	5.03	6.08	5.03	6.08	5.03	6.08	5.03	6.08	5.03
Sensitivity Analysis I	7.23	6.08	7.23	6.08	7.23	6.08	7.23	6.08	7.23	6.08	7.23	6.08
Sensitivity Analysis II	8.47	7.23	8.47	7.23	8.47	7.23	8.47	7.23	8.47	7.23	8.47	7.23
Sensitivity Analysis III	9.64	7.19	8.43	7.01	8.43	7.01	7.50	6.78	7.50	6.78	7.50	6.78

The results of the main analysis (including 95% confidence intervals) and side-by-side comparisons to each of the sensitivity analyses for motor vehicle collisions and non-motor vehicle collisions by age and sex is shown in Table 4. Since the RR for each of non-motor vehicle injuries is the same, they were grouped together for brevity in this table. Overall, the AAFs decrease with age and are significantly lower for women than men across all ages. Additionally, we can see that as binge drinking increases, the injury AAF also increases. For men and women, the sequential increase from 4 to 6 drinks per occasion showed relatively small corresponding increases in the AAF. However, when the NESARC data was used in the third sensitivity analysis, the jump to 8 or more drinks (seen in men aged 15-29) had a significant impact on the RR, resulting in a doubling of the AAF for motor vehicle collisions. Smaller relative increases were seen for non-motor vehicle collision AAFs at this age level, as well as overall for women and older age groups This is mirrored in Table 5 which shows the AAF increases within each injury subtype, but the amount of increase is augmented by the type of injury itself within non-motor vehicle collisions.

**Table 4:** Alcohol-attributable fractions and 95% confidence intervals for motor vehicle and non-motor vehicle collision - main analysis and each of the three sensitivity analyses.

	15-29				30-44				45-59				60-69				70-79				80+			
	M		W		M		W		M		W		M		W		M		W		M		W	
	AAF	±	AAF	±	AAF	±	AAF	±	AAF	±	AAF	±	AAF	±	AAF	±	AAF	±	AAF	±	AAF	±	AAF	±
**Main Analysis: Binge 4/5**																								
MVA	0.22	0.14	0.13	0.17	0.16	0.11	0.02	0.06	0.15	0.10	0.03	0.05	0.16	0.13	0.03	0.07	0.16	0.17	0.03	0.10	0.09	0.14	0.04	0.11
Non_MVA	0.43	0.21	0.26	0.22	0.30	0.15	0.01	0.00	0.32	0.16	0.03	0.01	0.34	0.20	0.04	0.01	0.32	0.25	0.03	0.02	0.12	0.15	0.03	0.01
																								
**Sensitivity Analysis I: Binge 5/6**																								
MVA	0.23	0.14	0.13	0.12	0.16	0.10	0.02	0.01	0.15	0.10	0.03	0.01	0.16	0.13	0.03	0.02	0.16	0.15	0.03	0.01	0.09	0.14	0.04	0.02
Non_MVA	0.45	0.20	0.27	0.22	0.32	0.15	0.02	0.01	0.33	0.15	0.03	0.01	0.35	0.21	0.04	0.01	0.33	0.23	0.03	0.02	0.12	0.15	0.03	0.01
																								
**Sensitivity Analysis I: Binge 6/7**																								
MVA	0.28	0.14	0.13	0.12	0.19	0.10	0.03	0.01	0.16	0.10	0.03	0.01	0.17	0.13	0.03	0.02	0.17	0.17	0.03	0.01	0.09	0.15	0.04	0.02
Non_MVA	0.47	0.21	0.27	0.23	0.33	0.15	0.02	0.01	0.33	0.15	0.03	0.01	0.35	0.21	0.04	0.01	0.33	0.25	0.03	0.02	0.12	0.17	0.03	0.01
																								
**Sensitivity Analysis III: NESARC data**																								
MVA	0.57	0.19	0.18	0.14	0.37	0.13	0.04	0.03	0.22	0.10	0.03	0.02	0.19	0.13	0.03	0.02	0.19	0.16	0.03	0.01	0.10	0.14	0.04	0.02
Non_MVA	0.52	0.22	0.28	0.23	0.35	0.15	0.02	0.01	0.34	0.15	0.04	0.01	0.35	0.21	0.04	0.01	0.33	0.23	0.03	0.02	0.12	0.15	0.03	0.01

**Table 5:** Alcohol-attributable fractions by injury subtype, men and women combined, for the main analysis and each of the sensitivity analyses.

Cause	Main Analysis			SA I			SA II			SA III		
	Total AAF	LB	UB	Total AAF	LB	UB	Total AAF	LB	UB	Total AAF	LB	UB
**Unintentional injuries**												
Motor vehicle collision	0.14	0.03	0.25	0.14	0.05	0.24	0.17	0.07	0.26	0.29	0.17	0.41
Poisonings	0.24	0.12	0.37	0.25	0.13	0.38	0.26	0.13	0.38	0.27	0.15	0.40
Falls	0.13	0.03	0.23	0.14	0.04	0.23	0.14	0.03	0.24	0.14	0.04	0.24
Fires	0.20	0.08	0.32	0.20	0.09	0.32	0.21	0.09	0.33	0.22	0.09	0.34
Poisonings and exposure to alcohol	1.00	1.00	1.00	1.00	1.00	1.00	1.00	1.00	1.00	1.00	1.00	1.00
Drowning	0.22	0.10	0.35	0.23	0.11	0.35	0.24	0.12	0.36	0.25	0.13	0.38
Other Unintentional injuries	0.27	0.13	0.40	0.27	0.14	0.41	0.28	0.15	0.42	0.30	0.16	0.44
**Intentional injuries**												
Self-inflicted injuries	0.27	0.13	0.41	0.28	0.14	0.42	0.29	0.14	0.43	0.30	0.16	0.45
Intentional self-poisoning by and exposure to alcohol	1.00	1.00	1.00	1.00	1.00	1.00	1.00	1.00	1.00	1.00	1.00	1.00
Homicide	0.28	0.14	0.43	0.29	0.15	0.44	0.30	0.16	0.45	0.32	0.17	0.48
Other intentional injuries	0.37	0.18	0.55	0.38	0.20	0.56	0.39	0.21	0.57	0.42	0.24	0.61

Motor vehicle collisions show the largest relative increases in AAF as alcohol consumption is increased, with the largest jump occurring for the third sensitivity analysis at over a 100% increase. Among non-motor vehicle collisions, the largest change in total AAF occurred both for homicide and other intentional injuries at about a 15% increase in the AAF from the lowest to the highest binge consumption scenarios.

## Discussion

We presented a novel method for computing AAFs for fatal injury using different inputs from many sources. The sensitivity analysis showed this method to be sensitive to increases in consumption over 100 grams per occasion particularly, which was apparent at the lower age groups where this consumption scenario occurred for men 15-29.

The highest impact factor in this calculation was the alcohol consumption variables, which in turn drive the relative risk function. However, the consumption variables in this analysis came from surveys (CADUMS for the main analysis, NESARC for the sensitivity analysis), which carries limitations with respect to reaching certain populations, and inherent biases in self-reported data that are common to survey instruments. Usual surveys are based on households, and populations such as institutionalized and homeless are not part of the sampling frame, particularly in telephone surveys. This has an effect for both methods since drinking distributions tend to be characterized by a "concentration of consumption". This means that a small portion of the population is likely to be responsible for a large proportion of the alcohol drinking. For instance, in the NESARC sample, the 6.7% of the heaviest White male drinkers consume 33% of the overall consumption, so excluding or undersampling relatively small groups may result in relatively large proportions of under coverage (see also [26]). Underreporting of consumption will also result in underestimation of the AAF since lower alcohol consumption would result in significantly lower relative risks, meaning that the computed AAF would be significantly reduced. Data around this topic is difficult to collect and the literature on this area is relatively sparse but tends to support the hypotheses that self-reports on alcohol consumption in medical epidemiology and in surveys are relatively valid overall [27-29]. However, some evidence shows that few questions about frequency of alcohol consumption embedded in health questionnaires yield higher levels of consumption compared to surveys where alcohol is the main topic [30]. Thus, for this analysis, the CADUMS data may be less reliable than for the NESARC data, but the discrepancy is difficult to quantify. Thus, more research is needed before one can further generalize on procedures on how to select the level of true consumption to be taken as basis for derive AAFs. Another limitation of this analysis was that the CADUMS survey also reported a low response rate, only 36.5%. However, the consequence of this would lead to an lower, and thus conservative, estimate since

Another limitation of this analysis is the fact that the same relative risk relationship was used for all non-motor vehicle collision deaths. While this was necessary given the available data in the literature in the original meta-analysis [20], there are almost certainly variations in risk for individual injury types. To further stabilize the risk functions and "parse out" individual risks for injury subtypes, more data points are needed to carry future meta-analyses, showing a need for more studies in this area.

An important point to discuss is why binge drinking had such a low impact on injury compared to average daily consumption, since most evidence points to heavy drinking leading to intoxication as the main mode of the incidence of alcohol-attributable injury [31-33].

The method to calculate the AAFs for binge drinking uses only a mean and, therefore, binge consumption is based on a point estimate. The method used to calculate the AAFs for average consumption uses a distribution and, therefore, average consumption calculates the relative risk for all levels of intake. We assume that the RR we calculated for binge drinking using a point estimate would be equal to the average RR we would obtain if we used a distribution approach; however, since the RR functions are not linear, these two estimates will never be equal. We are limited to using a point estimate method since the calculation of binge AAFs is based on knowledge we do not have of the distribution of binge consumption.

The Monte Carlo approach to derive confidence intervals was necessary, as there are no numeric derivations possible. It follows similar approaches in disease modeling and risk factor epidemiology (e.g., [34,35]).

## Conclusions

Overall, the described method included the main parameters known from the literature. Future research is necessary to refine the risk function, as there may be cultural differences in risk based on different environments (consider, e.g., the impact of highway safety on the impact of alcohol on highway fatalities). Similarly, the modeling of binge drinking distributions and potential interactions between binge drinking and average volume of alcohol consumption [36] may be improved based on new research. However, overall, the presented data allows for estimating the impact of alcohol consumption on traffic safety based on the best evidence to date, and should be used in new estimates of alcohol-attributable burden.

## Competing interests

The authors declare that they have no competing interests.

## Authors' contributions

BT participated in the design of the work, the conceptualization and development of it methods, some statistical analysis with respect binge risk and drafted the manuscript. KS performed the majority of statistical analysis and wrote selected sections of the methods. JR conceived of the study, participated in its design and coordination, and was involved in drafting the manuscript.

## Pre-publication history

The pre-publication history for this paper can be accessed here:

http://www.biomedcentral.com/1471-2458/11/265/prepub
